# Multiple cardiac complications due to sepsis and cardiac metastasis of a sarcomatoid urothelial cell carcinoma of the urinary bladder: A case report

**DOI:** 10.1016/j.ijscr.2025.111514

**Published:** 2025-06-14

**Authors:** Sjaak Pouwels, Wouter Fitski, Michael Magro, Yvette van der Linden-Foolen, Jeroen Stavast, Desiree H.C. Burger

**Affiliations:** a Department of Surgery, Bielefeld University – Campus Detmold, Klinikum Lippe, Detmold, NRW, Germany; b Department of Intensive Care Medicine, Elisabeth-Tweesteden Hospital, Tilburg, the Netherlands; c Department of Cardiology, Elisabeth-Tweesteden Hospital, Tilburg, the Netherlands; d Department of Pathology, Elisabeth-Tweesteden Hospital, Tilburg, the Netherlands

**Keywords:** Cardiac metastasis, Cardiac complications, Sarcomatoid urothelial cell carcinoma, Bladder cancer

## Abstract

**Introduction and importance:**

Of all malignancies, urinary bladder cancer is one of the most diagnosed ones. Majority of the patients with bladder cancer have a non-muscle invasive type that can be treated with conservative approaches. The sarcomatoid urothelial cell carcinoma is rare and aggressive tumour that can develop in the urinary bladder, ureter or kidney. Hereby, we present a patient that developed a cardiogenic shock due to a large cardiac metastasis of a sarcomatoid urothelial cell carcinoma.

**Case presentation:**

A seventy-one year old male was presented at our Emergency Department with complaints/ signs of dyspnoea, low blood pressure, tachycardia/atrial fibrillation, diarrhoea and general malaise. Trans Thoracic Echocardiography (TTE) was performed which showed a mass in the left ventricle suspect of malignant origin. After five days of hospital admission the patient died due to refractory shock. Autopsy reports showed extensive cardiac, spinal, peritoneal, peripheral fat and muscle tissue metastasis, due to the sarcomatoid urothelial cell carcinoma.

**Clinical discussion:**

Urothelial carcinomas are one of the most common urinary cancers of which sarcomatoid urothelial carcinoma is a rare, highly malignant and very aggressive type. The low incidence of this variant inhibits the conduct of randomised clinical trials and makes clinical management difficult.

**Conclusion:**

Due to its aggressive growth rate sarcomatoid urothelial cell carcinoma have a high probability of metastasizing, however cardiac metastasis are very rare.

## Highlights

• Of all malignancies, urinary bladder cancer is one of the most diagnosed ones.
• Majority of the patients with bladder cancer have a non-muscle invasive type.
• The sarcomatoid urothelial cell carcinoma is rare and aggressive tumour that can develop in the urinary bladder, ureter or kidney.
• The low incidence of this variant inhibits the conduct of randomised clinical trials.
• Due to its aggressive growth rate sarcomatoid urothelial cell carcinoma have a high probability of metastasizing

## 1 Introduction

Of all malignancies, cancer originating from the urinary bladder is one of the most diagnosed ones. The majority of patients with bladder cancer have a non-muscle invasive type that can be treated with conservative approaches. [1] The sarcomatoid urothelial cell carcinoma is a rare and aggressive tumour that can be present in the urinary bladder [2], ureter [3] or kidney [4,5]. This variant shows uncommon histological features, namely; 1) an epithelial component and 2) a non-epithelial sarcoma component.

The sarcomatoid type accounts for approximately 0.5 % of all urothelial carcinomas. [6] These sarcomatoid type bladder carcinomas have been associated with very rapid growth rate and are commonly already at an advanced stage at initial presentation. Patients usually present themselves with lower urinary tract symptoms and haematuria and management involves a multimodal approach. [3,5,7]

Pathological diagnosis is sometimes difficult to establish, even with the use of advanced histopathological and immunohistochemistry techniques. Unfortunately, due to its rarity limited information is available regarding treatment (e.g. chemotherapy) and prognosis. [[1], [2], [3], [4], [5], [6]]

Furthermore even less literature is available regarding the metastatic pattern of sarcomatoid urothelial cell carcinomas. Bone, lung, cutaneous, intestinal, liver and brain metastasis have been reported. [8] However cardiac metastasis due to sarcomatoid urothelial cell carcinomas have not yet been reported.

Hereby, we present a patient that had a sepsis and multiple cardiac complications due to a large cardiac metastasis of a sarcomatoid urothelial cell carcinoma, which was surgically removed three months earlier.

## 2 Case presentation

This case report has been reported according to the SCARE guidelines. [9] A seventy-one year old male was presented at our Emergency Department with complaints of dyspnoea on exertion, low blood pressure, tachycardia, diarrhoea and general malaise. He recently discontinued his diuretics due to hypotension. His medical history reported a non-ischemic cardiomyopathy with an estimated moderate left ventricular ejection fraction (LVEF) of 35–40 %, coronary artery disease with a stenosis of 50 % in the left anterior descending artery (LADA) and had suffered from several non-sustained ventricular tachycardias in the past. Secondly, approximately three months before, a cysto-prostatectomy with urostomy was performed because of a sarcomatoid urothelial cell carcinoma of the bladder. At initial presentation his blood pressure was 100/70 mmHg and he presented with a sinus tachycardia of 105 beats per minute. His respiratory rate was normal and his oxygen saturation was 100 %.

The initial laboratory tests showed an elevated high sensitive-Troponin of 0.127 ng/mL (reference values 0.00–0.030 ng/mL), N-terminal prohormone of Brain Natriuretic Peptide (NTproBNP) of 1595 pmol/L (reference values 0.0–27.0 pmol/L) and mildy elevated inflammatory parameters (C-reactive protein 40 mg/L (reference values 0–10 mg/L) and leucocyte count of 15.3*10^9^/L (reference values 4.0–10.0 *10^9^/L)). His chest x-ray showed no abnormalities. The initial diagnosis was thought to be a non-ST elevation myocardial infarction (NSTEMI) with a mild component of cardiac decompensation, based on the initial laboratory tests and electrocardiogram (ECG). The ECG showed ST depressions in lead I and lead V5. Therefore, the patient was admitted at the Coronary Care Unit (CCU) of our hospital and medical treatment was started involving intravenous diuretics, b-blockers and dual anti-platelet therapy.

**Fig. 1:**
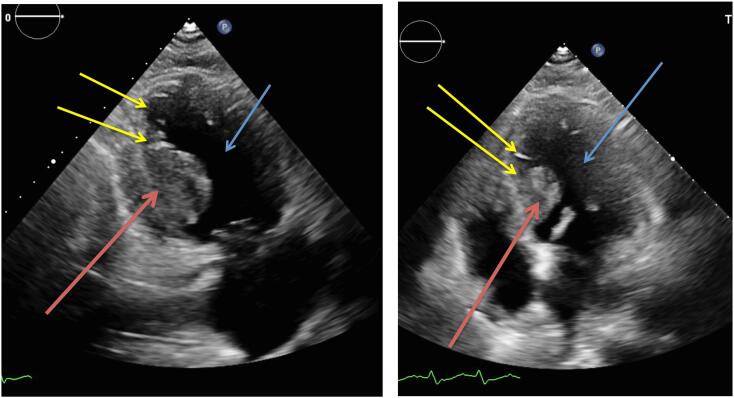
Trans Thoracic Echocardiography (TTE) images showing mass **(red arrows)** in the left ventricle **(blue arrows)** at the base of the left ventricular septum **(yellow arrows)** in the endocardium. (For interpretation of the references to colour in this figure legend, the reader is referred to the web version of this article.)

**Fig. 2:**
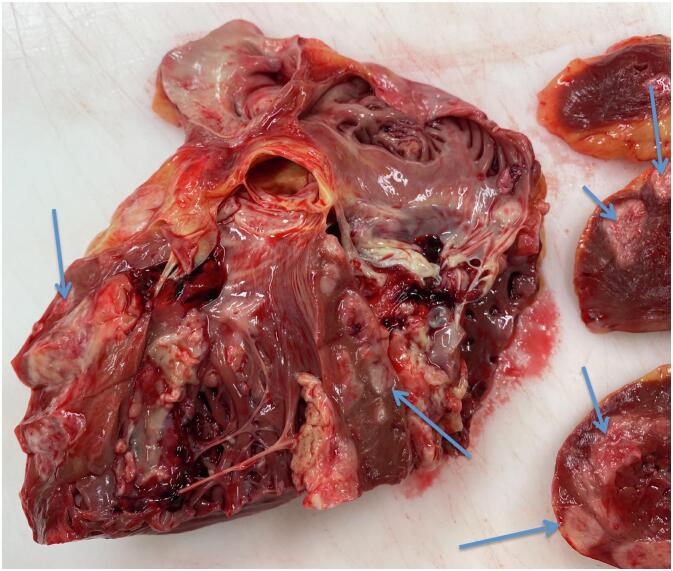
Macroscopic pathologic examination showing extensive metastases in the endocardium, myocardium and pericardium **(blue arrows)** in the myocardium. (For interpretation of the references to colour in this figure legend, the reader is referred to the web version of this article.)

**Fig. 3:**
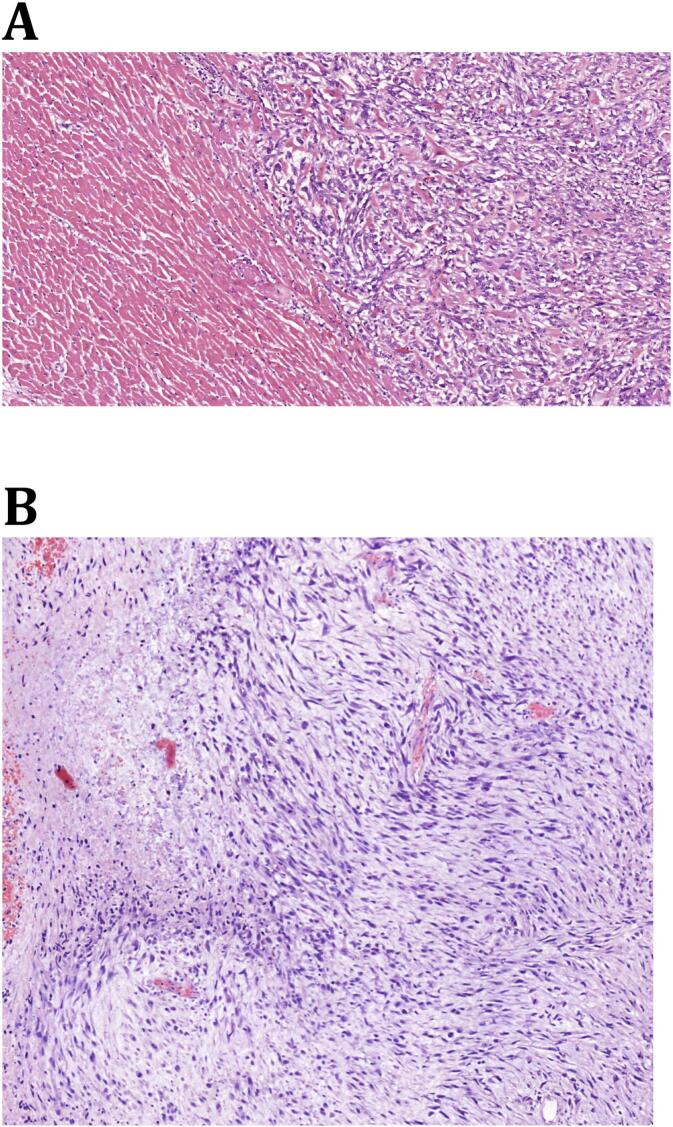
Microscopic evaluation of the cardiac metastasis due to a sarcomatoid urothelial cell carcinoma of the urinary bladder **(A)** compared with the original microscopic examination after the cystoprostectomy **(B).**

During admission a Trans Thoracic Echocardiography (TTE) was performed which showed a mass with a diameter of approximately 2–3 cm near the base of the left ventricular septum, suspect of malignant origin. (Fig. 1) It was thought to be in the endocardium. Interestingly this mass was not present on TTE images three months earlier. The same day he developed new onset atrial fibrillation followed by a third degree atrioventricular (AV) block involving an 8 s cardiac arrest. Therefore a temporary external pacemaker (PM) was inserted via the left femoral vein. Unfortunately, further investigation of the intraventricular mass involving a cardiac Magnetic Resonance Imaging (MRI) was delayed due to this external pacemaker. In the following days the patient developed a fever (39.5 degrees Celsius) and showed progression of inflammation parameters (CRP 82 mg/L and leucocyte count of 18.1* 10^9^/L). His blood cultures showed *Staphylococcus Capitis* for which he was treated with intravenous antibiotics. A few days later patient became respiratory insufficient due to cardiac decompensation caused by a ventricular tachycardia of approximately 240 beats per minute, which converted to sinus rhythm without assistance. He was transferred to the Intensive Care Unit (ICU) for Non-Invasive Mechanical Ventilation (NIV). Secondly noradrenalin was administered because of hypotension, due to a combined state of septic and cardiogenic shock. Due to a possible focus for his infection the external PM was removed. Once again a MRI was scheduled to investigate the mass in the left ventricle. Unfortunately the clinical course of the patient was getting so severe, after a total of five days of hospital admission the patient died due to refractory cardiogenic and/or septic shock. Autopsy reports (both macroscopic and microscopic evaluation) showed that there was extensive cardiac, spinal and peritoneal metastasis, due to the sarcomatoid urothelial cell carcinoma. Fig. 2 shows the extensive metastases in the endo-, myo- and pericardium and Fig. 3 shows the microscopic images. It needs to be stated that during the admission of the patient, we only found one mass on the TTE (as stated earlier) and during post-mortem pathological examination the earlier mentioned extensive metastasis was seen. Due to the rapidly deteriorating clinical situation of our patient, there was no possibility to do imaging studies (either Computed Tomography (CT) and/or Magnetic Resonance Imaging (MRI)).

## 3 Discussion

Urothelial carcinomas are one of the most common urinary cancers of which sarcomatoid urothelial carcinoma is a rare, highly malignant and a very aggressive type. [3,5,7] Frequent locations of this type of tumour are the urinary bladder, but in rare occasions also the ureter or the kidney. [[1], [2], [3], [4], [5], [6]] Due to its rarity very little is known about this type and even less is known about its metastatic behaviour. The only clinical information we have regarding metastatic patterns is from case reports showing metastasis to the bone, lung, cutaneous, intestinal, liver and brain. [8] Interestingly the majority of these studies were from or published in Asia.

To our knowledge, this is the first case that reports cardiac metastasis of a sarcomatoid urothelial cell carcinoma of the bladder. A previous case reported by Yamac et al. [10] reported about a peri-myocarditis, which was caused a metastasis of a less differentiated urothelial cell carcinoma (aT4, aN0, aMI, G3), with its origin in a large diverticle of the urinary bladder.

In our patient we found a mass in the left ventricle on TTE, which was suspect for a malignancy. (Fig. 1) Interestingly three months earlier this mass was not visible on the TTE. In terms of a differential diagnosis, three possible causes were taken into account, 1) primary cardiac tumour or 2) metastasis or 3) thrombus. It needs to be mentioned that primary cardiac tumours are exceedingly rare (incidence around 0.02 %) and secondly cardiac metastases are even rarer. In terms of primary cardiac tumours, myxomas are the most common (50–70 % of all cardiac tumours) and usually show very slow progression over several years. [11] Thirdly, in our patient the mass was located in the left ventricle, whereas 80 % of myxomas are found in the left atrium. [11] Regarding diagnostic modalities echocardiography remains the cornerstone in diagnosing cardiac tumours followed by further differentiating involving CT and MRI.

A more controversial issue is the pathophysiology of the sarcomatoid urothelial carcinoma, in particular its histogenesis. Two hypothesis are currently described in literature: 1) it is a collision tumour which means it is composed of two independent but simultaneously occurring epithelial and mesenchymal neoplasms. [4,5,7], 2) the sarcomatoid carcinoma has a same clonal origin but with branches that differentiate into different cell types. [4,5,7]

Sarcomatoid carcinoma is epithelial cancer that display cells with sarcomatoid morphological differentiation. [4,5,7] Both components express different kinds of tissue markers, namely cytokeratin (epithelial tissue marker) and vimentin (mesenchymal tissue marker). These markers co-exist at the same time. [4,5,7] The aetiology is currently unclear. The sarcomatoid carcinoma may be derived from monoclonal tumour cells and this might be associated with chromosome gene amplification and 9p21 chromosome deficiency. [4,5,7] With immunohistochemical techniques expression of the earlier mentioned vimentin and cytokeratin can be found. [5]

Clinically, patients usually present themselves with lower back pain and gross haematuria. This was also the case in our patient prior to initial surgery. Secondly, it has to be taken into account that in the majority of these patients the disease can progress asymptomatically, which results in a higher tumour stage (T2 or T3) and local (lymph) metastasis accompanied by renal insufficiency. [4,5,7] Our patient already had local lymph node metastases at initial presentation (lymph node in obturator fossa and aorto-iliac lymph node metastasis).

According to the guidelines of the European Association of Urology Guidelines radical surgery remains the gold standard for obtaining loco regional disease control, despite the low five-years overall survival (between 15 and 30 % per cent). [4,5,7]

## 4 Conclusion

Urothelial carcinomas are the most common urinary cancers of which sarcomatoid urothelial carcinoma is a rare, highly malignant and a very aggressive type. The low incidence of this variant inhibits the conduct of randomised clinical trials and makes clinical management difficult. Due to its aggressive growth rate, sarcomatoid urothelial cell carcinomas have a high probability of metastasizing of which cardiac metastases are very rare. In case of an unexplained mass in the heart with symptoms that cannot be adequately explained advanced imaging (cardiac CT or MRI) should be done.

## Consent to publish

Written informed consent was obtained from the patients' relatives for publication.

## Ethical approval

The medical ethical committee gave an exemption for this study, since it is a case report. Formal ethical approval therefore not required.

## Funding

None.

## Author contribution

**Collecting data:** Sjaak Pouwels, Wouter Fitski, Michael Magro, Yvette van der Linden-Foolen, Jeroen Stavast, Desiree H.C. Burger.

**Writing the manuscript:** Sjaak Pouwels, Wouter Fitski, Michael Magro, Yvette van der Linden-Foolen, Jeroen Stavast, Desiree H.C. Burger.

**Final approval:** Sjaak Pouwels, Wouter Fitski, Michael Magro, Yvette van der Linden-Foolen, Jeroen Stavast, Desiree H.C. Burger.

## Guarantor

Sjaak Pouwels.

## Research registration number

N/A.

## Clinical trial number

Not applicable.

## Conflict of interest statement

The authors have no conflict of interest to declare.

## Data Availability

Data is available upon reasonable request (please contact the corresponding author).
